# The War and University Studies

**Published:** 1914-08-29

**Authors:** Donald MacAlister

**Affiliations:** Principal. University of Glasgow


### The War and University Studies.

*To the Editor of* The Hospital.

Sir,—May I ask for your kind offices in conveying to undergraduate students called to active service for their country the assurance that the University of Glasgow will do what it can to safeguard their academic interests? The authorities whom I have been able to consult agree with me in recommending that to such students every consideration should be extended which the ordinances will permit.

In relation to attendance on courses of instruction, to duration of study, to periods of notice required, and the like, account will be taken of a student's absence on military duty, so as, if possible, to ensure that his graduation shall not be unduly delayed.—I am, Sir, yours very faithfully, (Signed)

Donald MacAlister, Principal.

University of Glasgow, August 19, 1914.

